# The efficacy of a short one-on-one nursing intervention in people with coronary heart disease: A randomized controlled trial protocol

**DOI:** 10.1097/MD.0000000000024405

**Published:** 2021-01-29

**Authors:** Wen Xia, Ting Liu, Jie Lei

**Affiliations:** a Department of Cardiovascular Medicine; b Physical Examination Center; c Department of Cardiovascular Medicine, Wuhan No.1 Hospital, Hubei, China.

**Keywords:** acute coronary syndrome, coronary heart disease, one-on-one nursing, protocol

## Abstract

**Background:**

To assess the efficacy of a short one-on-one nursing intervention in people with coronary heart disease (CHD).

**Methods:**

The experiment will be implemented from January 2021 to May 2021 at Wuhan No.1 Hospital. The experiment was granted through the Research Ethics Committee of Wuhan No.1 Hospital (W202012-8) and recorded in research registry (researchregistry6378). Patients are eligible for the study if they have a diagnosis of CHD, confirmed by their physician and lived independently. Exclusion criteria are:

1) complicating serious comorbidity such as a major psychiatric illness or chronic renal failure;
2) untreated malignancy or neurological disorder that impaired cognition;
3) major and uncorrected hearing loss. Patients who agree to participate in the study attend interviews at the hospital. Primary outcome is the acute coronary syndrome (ACS) response index, which has 3 separate scales for knowledge, attitudes and beliefs. Secondary outcomes are anxiety and depression measured by multiple affect adjective checklist. It consists of 132 alphabetically ordered adjectives that are either negative (e.g., fearful) or positive (e.g., joyful).

**Results:**

Table 1 reflects the comparison of the biochemical and clinical variables and the lifestyle factors.

**Conclusion:**

A relatively short education may increase knowledge, attitudes and beliefs about ACS and response to ACS symptoms in individuals with CHD.

**Trial registration number:**

researchregistry6378

## 1 Introduction

Although the mortality rate from ischemic heart disease has decreased in the past several decades in developed countries, it continues to cause approximately one third of all deaths in persons older than 35 years of age.^[1,2]^ It has been estimated that almost half of middle-aged men and one third of middle-aged women in the United States will have some symptoms of ischemic heart disease.^[3–5]^ Meanwhile, cardiovascular disease causes an estimated annual total of 4 million deaths in Europe and of 1.9 million deaths in the European Union, largely due to coronary heart disease (CHD), representing 47% and 40% of all deaths in Europe and the European Union, respectively.^[6,7]^ In Europe, cardiovascular disease leads to a total estimated annual cost of 196 billion euros of which approximately 54% is due to direct health care costs and 24% is due to lost productivity.^[8]^ Moreover, the effects of CHD are not limited to developed countries and it has been a serious social problem.

The largest contributor to delayed time to receipt of reperfusion for acute coronary syndrome (ACS) is patient delay in recognizing symptoms and deciding to seek treatment.^[9,10]^ Most studies of interventions to reduce delay in response to ACS symptoms have focused on mass media campaigns and have shown modest success at best. Previous studies have introduced one-on-one education performed by local health care providers for patients with cancer and showed improved outcomes.^[11,12]^ In an effort to reduce patient prehospital delay times, we conduct a randomized controlled trial protocol of a novel one-on-one education and counseling intervention designed for patients at risk for ACS, with the primary aim of promoting timely response to symptoms and reducing delay times. A secondary aim is to investigate the effect of the study intervention on knowledge, attitudes and beliefs about CHD and ACS symptoms.

## 2 Methods

The experiment will be implemented from January 2021 to May 2021 at Wuhan No.1 Hospital. The experiment was granted through the Research Ethics Committee of Wuhan No.1 Hospital (W202012–8) and recorded in research registry (researchregistry6378). Before the registration, the patients who are recruited receive written informed consent. Sequence of random numbers is generated by a computer. Sequentially numbered sealed opaque envelopes are used for the concealment of random numbers. All the patients participating in this study are randomly divided into control group and nursing group, with 35 patients in each group.

### 2.1 Inclusion and exclusion criteria

Patients are eligible for the study if they have a diagnosis of CHD, confirmed by their physician and lived independently. Exclusion criteria are:

1) complicating serious comorbidity such as a major psychiatric illness or chronic renal failure;
2) untreated malignancy or neurological disorder that impaired cognition;
3) major and uncorrected hearing loss. Patients who agree to participate in the study attend interviews at the hospital.

### 2.2 The intervention

Information-patients are given information about typical symptoms, possible variability in symptom presentation and the fact that onset may be gradual and intermittent, rather than classic sudden crushing chest pain.

Emotional component-patients are assisted in anticipating emotional responses to ACS symptoms and acknowledging that these responses can delay the receipt of treatment. They are told that denial or suppression of the serious nature of symptoms is common but contributes to treatment delay and that attribution of symptoms to a body system other than the heart is common. Emotional issues are addressed partially through the use of scenarios featuring individuals who resembled the patient. Through roleplaying, patients are asked to anticipate emotions they may have when they experience symptoms of ACS and guide through the appropriate action steps.

Social factors-patients are advised to consult immediately with their spouse in the case of symptoms. When present with the patient, the spouse or family member is invited to attend the intervention session and are “deputized” to act as the decision maker if the patient hesitates to call the emergency number within 15 minutes of symptom onset.

The intervention is delivered in an individualized, one-on-one session of approximately 40 minutes with the patient. Using a script, an experienced cardiovascular nurse uses a flipchart with the main points listed and pictures illustrating the process of coronary occlusion and how reperfusion therapies restore blood flow to the myocardium. Patients are asked to place it in a prominent place in the home.

### 2.3 Outcomes

Primary outcome is the ACS response index,^[13]^ which has 3 separate scales for knowledge, attitudes, and beliefs. Secondary outcomes are anxiety and depression measured by multiple affect adjective checklist. It consists of 132 alphabetically ordered adjectives that are either negative (e.g., fearful) or positive (e.g., joyful).

### 2.4 Statistical analysis

Through utilizing the Microsoft Excel 2013, the data is recorded, and the analysis of all the data is carried out through utilizing IBM SPSS Statistics for Windows, version 20 (IBM Corp., Armonk, NY). With the χ2-tests and independent t-tests, the analysis for continuous variables and categorical variables are implemented respectively. All data are described with proper features, such as percentage, average and median. *P* value less than .05 indicates that there is statistical significance.

## 3 Results

Table 1 reflects the comparison of ACS Response Index scores for knowledge, attitudes and beliefs at study entry, 3 months and 12 months.

**Table 1:** Comparison of ACS response index scores for knowledge, attitudes and beliefs at study entry, 3 months and 12 months.

	Study group (n = 35)	Control group (n = 35)	
Variables	Mean+SD	Mean+SD	*P* value
Knowledge			
Baseline			
3 mo			
12 mo			
Attitudes			
Baseline			
3 mo			
12 mo			
Beliefs			
Baseline			
3 mo			
12 mo			

ACS = acute coronary syndrome, SD = standard deviation.

## 4 Discussion

CHD remains a significant public health problem in the developed world.^[14,15]^ Recently, the official statistics office of the American Heart Association estimated that about 15.4 million persons older than 20 years in the United States have ischemic heart disease.^[16]^ For ACS, the prevalence rate was estimated to be 2.9%. The effect of an ACS on mortality is largely dependent on the time from symptom onset to the time of reperfusion.^[17]^ Reperfusion therapy with either percutaneous coronary intervention or fibrinolytic drugs leads to lower mortality and fewer complications.^[18]^ Maximum benefit is achieved when reperfusion is performed in ACS within 60 minutes of symptom onset. The benefit from both reperfusion techniques decreases markedly if they are received more than 3 hours after symptom onset, although there may be some benefit up to 12 hours after symptoms start. Better knowledge of ACS symptoms has sometimes been found to be associated with shorter prehospital delay times.^[19]^ Enhancing knowledge must be considered an essential first step to promoting appropriate patient behavior in responding to ACS symptoms. Currently, few interventions have used one-on-one delivery of the information and few investigators have evaluated intermediate outcomes that potentially influence patient treatment-seeking behavior in response to ACS symptoms.

## 5 Conclusion

A relatively short education may increase knowledge, attitudes and beliefs about ACS and response to ACS symptoms in individuals with CHD.

## Author contributions

Jie Lei designs the protocol. Ting Liu reviews the protocol and performs the data collection. Wen Xia finishes the manuscript. All of the authors approved the submission.

**Conceptualization:** Wen Xia.

**Data curation:** Wen Xia.

**Formal analysis:** Ting Liu.

**Funding acquisition:** Jie Lei.

**Investigation:** Ting Liu.

**Methodology:** Ting Liu.

**Writing – original draft:** Wen Xia.

## Abbreviations

Abbreviations: ACS = acute coronary syndrome, CHD = coronary heart disease.
